# Clinical Characteristics of Complex Karyotype Soft Tissue Sarcomas: A Single-Institution Cohort Study

**DOI:** 10.3390/medicina62020271

**Published:** 2026-01-27

**Authors:** Eun-Young Lee, June Hyuk Kim, Jong Woong Park, Hyun Guy Kang, Seog-Yun Park, Jiyu Sun, Seo-Young Kim, Ahyoung Cho, Bora Lee, Hye Jin You

**Affiliations:** 1 Cancer Microenvironment Branch, Division of Cancer Biology, Research Institute, National Cancer Center, Goyang 10408, Republic of Korea; eylee@ncc.re.kr (E.-Y.L.); 76961@ncc.re.kr (A.C.); 2 Department of Orthopaedic Surgery, Center for Rare Cancer Center, National Cancer Center, Goyang 10408, Republic of Korea; docjune@ncc.re.kr (J.H.K.); jwpark82@ncc.re.kr (J.W.P.); ostumor@ncc.re.kr (H.G.K.); 73758@ncc.re.kr (B.L.); 3 Rare and Pediatric Cancer Branch, Division of Rare and Refractory Cancer, Research Institute, National Cancer Center, Goyang 10408, Republic of Korea; 4 Department of Cancer Biomedical Science, National Cancer Center Graduate School of Cancer Science and Policy, Goyang 10408, Republic of Korea; 5 Department of Public Health and AI, National Cancer Center Graduate School of Cancer Science and Policy, Goyang 10408, Republic of Korea; jiyusun@ncc.re.kr; 6 Department of Pathology, National Cancer Center Hospital, National Cancer Center, Goyang 10408, Republic of Korea; 11740@ncc.re.kr; 7 Integrated Biostatistics Branch, Division of Cancer Data Science, Research Institute, National Cancer Center, Goyang 10408, Republic of Korea; 8 Biostatistics Collaboration Team, Research Institute, National Cancer Center, Goyang 10408, Republic of Korea; sykim24@ncc.re.kr

**Keywords:** complex karyotype soft tissue sarcoma, undifferentiated sarcoma, FNCLCC grade, co-morbidity

## Abstract

*Background and Objectives*: This study aimed to describe the clinical characteristics and survival outcomes of three representative complex karyotype soft tissue sarcoma (STS) subtypes—undifferentiated sarcoma (US, primarily undifferentiated pleomorphic sarcoma (UPS)), myxofibrosarcoma (MFS), and leiomyosarcoma of soft tissue (LMS-ST)—using data from a single-institution cohort. *Materials and Methods*: A retrospective review of 124 patients treated at a single tertiary referral center between 2002 and 2024 was conducted. Clinicopathologic characteristics and survival outcomes were analyzed. Kaplan–Meier methods were used to estimate overall survival (OS). Cox proportional hazards regression models were applied to identify independent prognostic factors for survival, incorporating variables such as age, sex, tumor stage, and treatment modality. *Results*: The cohort comprised 36 cases of US, 64 of MFS, and 24 of LMS-ST. OS and survival after cohort enrollment (S-NCC) were evaluated both by subtype and across the entire cohort to assess potential differences across tumor subgroups. In both univariable and multivariable analyses, US subtypes showed poorer survival than MFS and LMS-ST. FNCLCC grade 3 emerged as a significant adverse prognostic factor for survival across all three subtypes. For FNCLCC grade 3 patients, the presence of benign prostatic hyperplasia (BPH) was significantly associated with an increased risk of death. *Conclusions*: Among the three subtypes, US demonstrated the most aggressive clinical course, MFS was notable for frequent local recurrence but relatively favorable survival, and LMS-ST showed intermediate outcomes. These findings highlight the clinical heterogeneity of complex karyotype STS and provide a foundation for future studies integrating molecular and multi-omics data to refine risk stratification and therapeutic strategies.

## 1. Introduction

Soft tissue sarcomas (STSs) are rare malignant tumors, accounting for approximately 0.7–1% of all adult cancers [1]. They encompass more than 70 histologic subtypes and exhibit marked heterogeneity in clinical behavior and prognostic outcomes [1,2]. In Korea, previous reviews have emphasized that STSs represent a highly heterogeneous group of diseases and that management in specialized centers is essential because of their diagnostic and therapeutic complexity [3,4]. Diagnosis for STS is primarily based on histopathological and cytogenetic analyses. STSs can be broadly classified into two categories according to their karyotypic characteristics. The first includes tumors with relatively simple karyotypes harboring specific recurrent genetic alterations, most commonly oncogenic gene fusions, such as EWSR1–FLI1 in Ewing sarcoma, FUS–DDIT3 in myxoid liposarcoma, and SS18–SSX1/2 in synovial sarcoma [5,6,7,8,9]. The second category comprises STSs with complex karyotypes, characterized by multiple chromosomal abnormalities without a specific recurrent genetic pattern [5,6,7,8,9,10]. Among these, undifferentiated sarcoma (US), myxofibrosarcoma (MFS), and leiomyosarcoma of soft tissue (LMS-ST) are representative subtypes characterized by complex karyotypes, each demonstrating distinct clinical features and prognostic patterns [5,6,8,9,11,12,13,14]. Given their overlapping clinical presentations but variable therapeutic responses, subtype-specific analysis is crucial for optimizing patient management.

Approximately 20% of STSs are classified as tumors of uncertain differentiation according to the fifth edition of the World Health Organization Classification of Soft Tissue and Bone Tumors [1]. This category includes undifferentiated pleomorphic sarcoma (UPS), undifferentiated spindle cell sarcoma (USS), undifferentiated round cell sarcoma (URS), and other undifferentiated types. These tumors typically exhibit aggressive clinical behavior, with higher rates of distant metastasis and poorer survival following relapse [1,2,15,16]. Genomic characteristics classify US into five rearrangement signatures (USARC.RS 1, 2, 3, 4 and 5) and are considered as a representative example of chromothripsis [15], in which unclassified sarcomas are included, characterized as spindle, round cell, epithelioid or pleomorphic: complex karyotype STSs. MFS, in contrast, frequently arises in the extremities of older adults and is characterized by a high rate of local recurrence despite wide surgical excision, posing persistent challenges for locoregional control [17,18,19,20]. LMS-ST, which originates from non-uterine or vascular smooth muscle elements, differs biologically from uterine leiomyosarcoma. Once metastasis occurs, LMS-ST often demonstrates limited response to systemic therapy [21,22,23]. The female predominance seen in uterine LMS is less consistent in LMS-ST, and anatomical heterogeneity can lead to variable outcomes.

Although these subtypes share complex genomic alterations, their biological and clinical behaviors differ markedly. Given their rarity, assembling sufficiently large, histologically validated cohorts for each subtype remains a significant challenge—even in tertiary centers. Consequently, analyses combining US, MFS, and LMS-ST within a single institutional cohort are essential to achieve meaningful statistical power and to facilitate comparative interpretation under consistent diagnostic and therapeutic conditions.

In Korea, no prior large-scale clinical study has comprehensively evaluated these three subtypes within one unified dataset. Most domestic reports have addressed STSs as a heterogeneous group or focused primarily on treatment modalities, such as limb-salvage surgery in extremity STSs, rather than on subtype-specific prognostic outcomes [3,4]. This highlights the urgent need to establish and share well-annotated clinical cohort to better understand these sarcoma subtypes.

Several studies have suggested similarities in clinical behavior or genetic aberrations among UPS, MFS, and LMS, all of which are classified as complex karyotype STSs [12,13,14]. However, it remains unclear whether these similarities are sufficiently robust to justify combining these subtypes for therapeutic strategies or clinical characterization.

Therefore, this study aims to characterize the clinicopathologic features and survival outcomes of adult patients with UPS, MFS, and LMS-ST treated at a single tertiary referral center in Korea. By analyzing these three representative complex karyotype STSs as a combined cohort while simultaneously performing subtype-specific comparisons, this study provides valuable real-world data to enhance clinical understanding and establish a foundation for future multi-omics integration. This approach addresses the limitations imposed by the rarity of individual complex karyotype STS subtypes by integrating clinical characteristics across multiple rare entities while preserving subtype-specific analyses, thereby enabling statistically meaningful evaluation to inform subsequent studies.

## 2. Materials and Methods

### 2.1. Ethics Approval and Consent to Participate

This study was approved by the Ethics Review Board of the National Cancer Center (NCC), Korea (IRB No. NCC2017-0062; approved on 8 March 2017) for both retrospective and prospective analyses. Written informed consent was obtained from all patients prior to surgery, and all samples were collected and stored in accordance with the principles of the Declaration of Helsinki. The clinicopathologic characteristics of the patients and tumors are summarized in Table 1 and Supplementary Table S1.

### 2.2. Patient Cohort and Study Design

We retrospectively reviewed the medical records of 124 patients diagnosed with US, MFS, or LMS-ST between 2002 and 2024 at a single tertiary referral center. An initial pilot study (*n* = 42), comprising patients diagnosed and treated surgically before 2018, was conducted to identify complex karyotype STS subtypes associated with poor prognosis and unmet therapeutic needs. This pilot cohort was selected based on the availability of archived tumor specimens from the NCC biobank.

Based on these findings, a subsequent main study (*n* = 82) was performed to expand the analysis of selected complex karyotype STSs. Some patients had undergone prior surgery with the same diagnosis before enrollment in this cohort, and their medical records were retrospectively reviewed. Accordingly, all patients were combined into a single cohort to enable comprehensive analysis of clinical outcomes beyond surgical tissue characteristics.

### 2.3. Inclusion Criteria and Pathologic Review

Inclusion criteria were those aged ≥ 23 years, histological confirmation of complex karyotype STS, and availability of comprehensive clinical and follow-up data (Table 1). All pathological specimens were centrally reviewed to ensure consistent and accurate subtype classification.

Histologic diagnoses were established according to the standard NCC diagnostic workflow by expert sarcoma pathologists at the NCC Hospital, incorporating clinical information, morphologic assessment, and molecular analyses. Complex karyotype STSs were not defined by specific cytogenetic breakpoints; instead, a histopathology-based classification was applied. Accordingly, US (including UPS, URS, and USS), MFS, and LMS-ST were included, as these subtypes are widely recognized as representative sarcomas characterized by complex, non-recurrent genomic alterations [5,7,8,9,10,11,12,13,14,24,25].

### 2.4. Clinical Variables

Collected variables included age, sex, Body mass index (BMI), primary tumor site and ICD-10 code, histological subtype and tumor grade according to Fédération Nationale des Centres de Lutte Contre Le Cancer (the French Federation of Cancer Centers Sarcoma Group, FNCLCC) histologic grading systems (grade1/2/3) [24,25,26], clinical presentation (primary, recurrent, or metastatic disease at diagnosis), treatment modality (surgical resection, radiotherapy, chemotherapy), comorbidities, and dates of diagnosis, treatment, recurrence, last follow-up, or death.

### 2.5. Statistical Analysis

Overall survival (OS) was defined as the interval between the date of the first diagnosis and death from any cause or the date of last follow-up (censored). Survival after cohort enrollment (S-NCC) was defined as the interval from the surgery at the time of enrollment in this cohort to death from any cause or the date of last follow-up (censored). Survival curves were estimated using the Kaplan–Meier method and compared using the log-rank test. Univariable and multivariable Cox proportional hazards models were applied to evaluate the associations between clinical variables—including subtype, grade, and clinical presentation—and OS and S-NCC. Among the prespecified covariates, BMI was the only variable with missing data, whereas no missingness was observed for the remaining clinical and pathological variables. Firth’s penalized likelihood approach was applied where appropriate to reduce bias associated with small sample sizes or rare events. All statistical analyses were conducted using R version 4.4.1 and SAS software version 9.4 (SAS Institute Inc., Cary, NC, USA).

## 3. Results

### 3.1. Cohort Characteristics

The pilot study (*n* = 42) identified complex karyotype soft tissue sarcomas STSs as priority subtypes associated with poor prognosis and unmet therapeutic needs. Based on these findings, the cohort was expanded to include an additional 82 patients. Patients with prior surgery for the same diagnosis were included through retrospective review of clinical records, resulting in a final combined cohort for comprehensive analysis of clinical outcomes.

A total of 124 patients were included in the analysis: 36 with US, 64 with MFS, and 24 with LMS-ST (Table 1). The median age at diagnosis was 60 years (interquartile range (IQR), 51–70.25), consistent with previously reported national and international cohorts. The sex distribution was balanced overall (56% male, 44% female). At diagnosis, 75% of patients presented with primary disease, 19% with recurrent disease, and 6% with metastatic disease (Table 2). FNCLCC grade 3 tumors accounted for 58% of the cohort. Female predominance was observed in LMS-ST, whereas US and MFS were more common in males. High-grade tumors were most frequent in US (91.7%), compared with LMS-ST (58.3%) and MFS (39.1%).

### 3.2. Survival Outcomes in Complex Karyotype STSs

**Figure 1:**
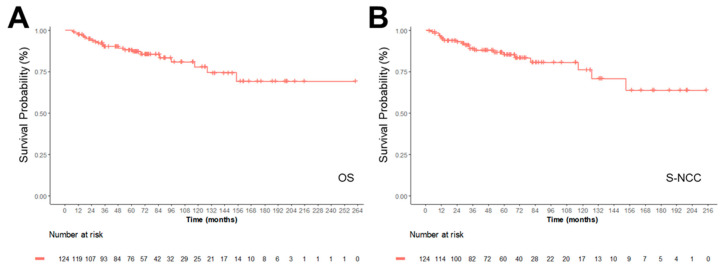
Survival probability of Complex Karyotype STS Cohort. Overall survival (OS, (**A**)) and Survival after NCC cohort enrollment (S-NCC, Surgery, (**B**)) were shown as Kaplan-Meier curves, with survival time expressed in months.

**Figure 2:**
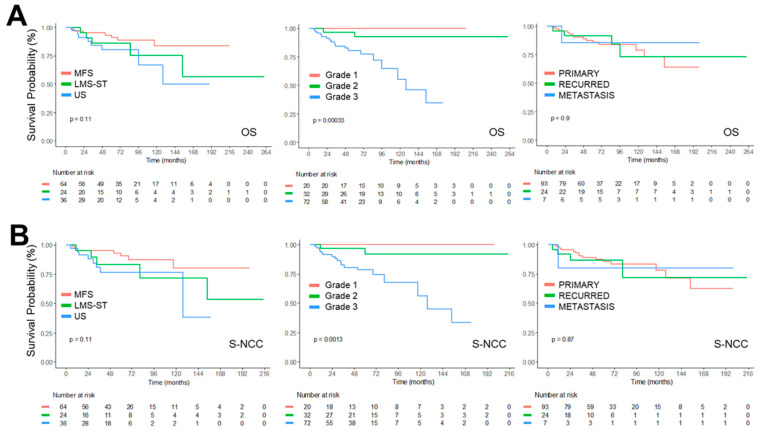
Kaplan–Meier survival plots for OS and S-NCC of patients with Complex Karyotype STSs. (**A**) Kaplan–Meier survival plots of OS were expressed according to subtypes (**left**), FNCLCC Grades (**middle**), and Presentations (**right**) as graphs. (**B**) Kaplan–Meier survival plots of OS were expressed according to subtypes (**left**), FNCLCC Grades (**middle**), and Presentations (**right**) as graphs. Red-, Green- and Blue-colored lines in each graph were annotated.

The OS rates at 1, 3, 5, and 10 years in this cohort were 97.6%, 90.5%, 88.3%, and 77.9%, respectively (Figure 1, Table 3). Since some patients have surgical treatment before enrollment in this cohort, Survival after cohort enrollment (S-NCC), was analyzed, which was similar to OS. US subtypes were associated with the worst Survival (Figure 2).

### 3.3. Risk Factors for Survival Outcomes in Complex Karyotype STSs

To identify clinical factors significantly associated with survival, we performed a multivariable Cox proportional hazards regression analysis with variables that showed statistical significance in the univariable analysis. Interestingly, FNCLCC grade 3 was identified as an independent predictor of OS (HR, 10.87, *p* = 0.0024) as well as S-NCC (HR, 8.29, *p* = 0071) (Table 4, Figure 2).

A significant difference was revealed between MFS and US in OS (HR, 2.79, *p* = 0.0488) and S-NCC (HR, 2.86, *p* = 0.045), whereas comparisons involving LMS-ST were not statistically significant. No significant difference was observed between LMS-ST and MFS in OS (HR, 2.14, *p* = 0.1938) and S-NCC (HR, 2.05, *p* = 0.224). Some patients underwent radiotherapy or adjuvant chemotherapy, which did not confer a significant survival benefit according to Cox regression analysis (Table 4).

**Figure 3:**
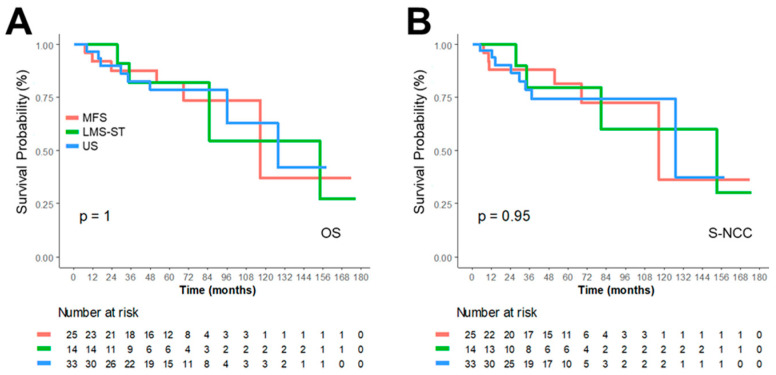
Kaplan–Meier survival curves for OS and S-NCC in patients with Complex Karyotype STSs Grade 3. Subtype-specific Survival is shown for OS (**A**) and S-NCC (**B**) as a function of time (months). The red line represents MFS, the green line LMS-ST, and the blue line US.

Cox regression analysis further demonstrated a strong association between survival and FNCLCC grade (Table 4, Figure 2), prompting additional subgroup analyses among patients with FNCLCC grade 3 tumors (Table 5). This subgroup included 72 patients (US = 33, LMS-ST = 14, MFS = 25). Within this high-grade cohort, tumor subtype was not significantly associated with prognosis according to univariable Cox regression (Table 5, Figure 3), suggesting shared biological behavior among high-grade complex karyotype STSs. Similarly, tumor presentation (primary vs. recurrent/metastatic) was not significantly correlated with prognosis in FNCLCC grade 3 patients.

### 3.4. Co-Morbidities and Survival Outcomes in Complex Karyotype STSs

**Figure 4:**
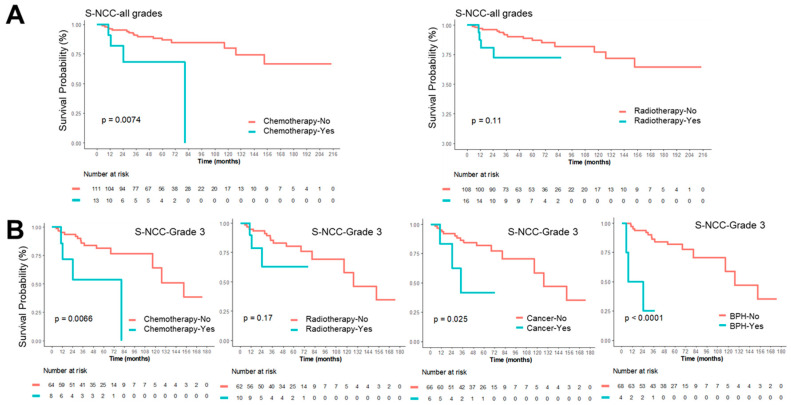
Kaplan–Meier survival plots of S-NCC for patients with complex karyotype STSs and comorbidities. (**A**) Patients of all tumor grades stratified by receipt of chemotherapy (left) and radiotherapy (right), shown as a function of survival time (months). (**B**) Grade 3 patients stratified by chemotherapy, radiotherapy, and the presence of cancer or BPH. Red and blue lines in each graph indicate the respective comparison groups.

This cohort consists exclusively of adult patients with complex karyotype STSs, who may have a higher likelihood of developing age-related or metabolic co-morbidities such as diabetes mellitus (DM), hypertension (HT), obesity, or secondary malignancies compared with younger populations. Therefore, we investigated whether any co-morbid conditions were associated with prognosis in the FNCLCC grade 3 patient subgroup (Table 5, Figure 4). Among these patients, several co-morbidities were observed, including DM, HT, tuberculosis (TB), other cancers (both sarcomatous and non-sarcomatous), and benign prostatic hyperplasia (BPH). BPH was unexpected independent risk factor for poorer survival among grade 3 patients, though small numbers warrant cautious interpretation. Neither BMI, DM, nor HT showed statistically significant associations with survival in either univariable or multivariable Cox regression analyses (Table 4 and Table 5). These findings indicate that metabolic comorbidities do not independently impact prognosis in CKS STSs.

## 4. Discussion

This cohort study provides a detailed description of the clinical features and outcomes of 124 patients with three major complex karyotype STS subtypes (US, MFS, and LMS-ST) treated at a single tertiary cancer center in Korea. To our knowledge, this is among the first reports in Korea to systematically compare these three entities within the same institutional framework, thereby minimizing variability in diagnostic approach and treatment protocols.

Our analysis confirmed several distinctions among the subtypes. US was associated with older age, a markedly high proportion of high-grade tumors, and the poorest outcomes in terms of both OS and S-NCC, consistent with international data [1,2]. MFS exhibited the highest frequency of local recurrence, reflecting its infiltrative growth pattern [17,19,20], yet OS outcomes were relatively favorable. LMS-ST showed intermediate results, with female predominance and biological differences from uterine LMS, consistent with previous report between uterine and extra-uterine LMS [6,19,20].

This cohort underscores the substantial heterogeneity that exists even within the “complex karyotype” group of STSs. Although US, MFS, and LMS-ST share extensive genomic instability rather than recurrent gene fusions, their distinct clinical trajectories indicate that histology alone cannot fully explain their biological diversity. However, the rarity of these tumors has delayed progress in understanding their underlying mechanisms.

In this study, we demonstrated that FNCLCC grade 3 tumors across these subtypes exhibit comparable prognostic outcomes, supporting the rationale for analyzing them collectively when developing therapeutic strategies for aggressive complex karyotype STSs.

Because of the limited number of cases in some histologic subtypes (especially LMS-ST), we combined subtypes in selected analyses to increase statistical stability. However, we acknowledge that this strategy may mask clinically meaningful heterogeneity across subtypes, and future studies with larger sample sizes are warranted to permit adequately powered subgroup analyses. Furthermore, we performed a sensitivity multivariable Cox regression analysis adjusting for calendar time by including an era indicator (prospective-only cohort vs. combined retrospective and prospective cohort) as a covariate. The era variable was not significantly associated with overall survival (hazard ratio [HR] = 0.82; 95% confidence interval [CI], 0.33–2.03; *p* = 0.661). Importantly, the main conclusions remained unchanged compared with the primary multivariable models (Table 4 and Supplementary Table S2), supporting the robustness and reliability of the combined cohort analysis.

Interestingly, univariable Cox regression assessing the influence of individual comorbidities on survival outcomes revealed that BPH was an unexpected independent predictor of poorer survival in grade 3 patients in OS as well as S-NCC; however, this finding should be interpreted cautiously given the limited number of affected cases (Table 5 and Figure 4). Both univariable and multivariable Cox regression analyses did not show any significant association of BMI, DM, and HT with OS as well as S-NCC (Table 4 and Table 5), indicating that metabolic comorbidities are not independent prognostic factors in complex karyotype STSs.

In the prostate, proinflammatory processes have been shown to promote hyperplastic changes that contribute to the development of BPH. Chronic inflammation in the aging prostate creates a microenvironment that facilitates cellular proliferation and hypertrophy, thereby supporting BPH progression [27,28]. Moreover, BPH has been considered a potential risk factor for prostate cancer, particularly in the presence of concomitant prostatitis or sustained inflammatory signaling [29,30,31]. These observations highlight the close interplay between chronic inflammation, benign prostatic disease, and malignant transformation. In the context of chronic inflammatory conditions, BPH has also been shown to be closely associated with systemic immune–inflammation indices in elderly populations [32,33,34]. Given that the majority of patients in our cohort were elderly, systemic inflammatory status may represent a relevant confounding factor. However, in the present study, we did not systematically evaluate comorbid conditions such as urinary tract infections or other inflammatory disorders. Accordingly, this finding should be interpreted with caution. Future studies incorporating detailed comorbidity profiling may provide important insights into complex clinical presentations and the progression of complex karyotype STSs.

Clinical datasets such as ours serve as an essential foundation for future molecular and translational investigations. The translational implications are significant. Large-scale initiatives such as TCGA and CPTAC have demonstrated that multi-omics approaches can identify molecular subtypes, prognostic biomarkers, and therapeutic targets in sarcoma [35,36]. Proteogenomic profiling suggests that protein expression modules may stratify risk independent of histology, while transcriptomic studies point to a continuum between MFS and US. Our well-annotated clinical cohort provides an essential foundation to connect such molecular features to prognosis in Korean patients. Furthermore, immune profiling studies have suggested that US and MFS may harbor subsets responsive to immune checkpoint blockade [37], while LMS-ST has traditionally been considered “immune-cold” yet contains rare immune-inflamed subgroups [38]. These observations underscore the potential of combining clinical cohorts with molecular and immunological datasets to refine patient selection for emerging therapies.

In the present study, preoperative radiotherapy and chemotherapy were associated with poorer prognosis (Table 4). Nonetheless, this observation should be interpreted with caution given the limited number of treated cases (radiotherapy: n = 16; adjuvant chemotherapy: n = 13), the potential for selection bias, and the lack of detailed documentation regarding treatment response. As standardized guidelines for preoperative therapy are lacking, such interventions are typically reserved for high-grade, stage 3, or otherwise non-salvageable sarcomas following multidisciplinary evaluation. Consequently, the observed associations may reflect underlying disease severity rather than true treatment effects, limiting the generalizability of these findings. Further research involving larger, treatment-specific cohorts is needed to clarify the true impact of perioperative therapy on outcomes in complex karyotype STSs.

Limitations of this study include the modest sample size, particularly for LMS-ST, and the retrospective design. Due to the limited sample size and number of events (deaths), we were unable to perform a multivariable survival analysis for FNCLCC grade 3; therefore, only the univariable results are presented. Variability in treatment regimens across two decades may also have influenced outcomes, and molecular data were not integrated. Nonetheless, this cohort provides valuable baseline evidence and underscores the importance of combining clinical and molecular approaches.

## 5. Conclusions

US demonstrated the most aggressive clinical course, MFS had recurred locally frequently but relatively favorable OS, and LMS-ST displayed intermediate outcomes. By capturing these distinctions within a Korean cohort, our study provides a reference point for both clinical practice and translational research. Future multicenter collaborations that integrate multi-omics and immunological profiling with detailed clinical annotation will be critical to refine risk stratification and advance personalized treatment strategies for patients with complex karyotype STSs.

## Figures and Tables

**Table 1:** Summary of clinical and pathologic information.

Total Number of Individuals	124
Age (years)	
Median (IQR)	60 (51–70.25)
<40	13 (10.5%)
40–49	14 (11.3%)
50–59	33 (26.6%)
60–69	27 (21.8%)
70–79	25 (20.2%)
more than 80	12 (9.7%)
Sex	
Male	69 (55.6%)
Female	55 (44.4%)
Anatomical site	
Anatomical wall	3 (2.4%)
Back	9 (7.3%)
Chest wall	7 (5.6%)
Long bones of lower limb	2 (1.6%)
Lower limb	63 (50.8%)
Pelvis	12 (9.7%)
Upper limb	28 (22.6%)
Subtypes	
US	36 (29.0%)
UPS *	26
USS	7
URS	1
UES	1
US	1
LMS-ST **	24 (19.4%)
MFS	64 (51.6%)
FNCLCC GRADE	
3	72 (58.1%)
2	32 (25.8%)
1	20 (16.1%)
Presentation	
Metastasis	7 (5.6%)
Recurred	24 (19.4%)
Primary	93 (75.0%)
BMI ***	
>25	45 (36.3%)
≤25	78 (62.9%)
Radiotherapy	
Yes	16 (12.9%)
No	108 (87.1%)
Chemotherapy	
Yes	13 (10.5%)
No	111 (89.5%)

Values are presented as number (%) or median (range). IQR, Interquartile range; US, undifferentiated sarcoma; UPS, undifferentiated pleomorphic sarcoma; USS, undifferentiated spindle cell sarcoma; URS, undifferentiated round cell sarcoma; UES, undifferentiated epithlioid sarcoma; LMS-ST, leiomyosarcoma-soft tissue; MFS, myxofibrosarcoma; BMI, body mass index; FNCLCC, Fédération Nationale des Centres de Lutte Contre Le Cancer. * Two patients diagnosed with primary leiomyosarcoma of bone were identified. ** One patient with primary UPS of bone was included. *** BMI missing 1 patient.

**Table 2:** Subtype-type specific characteristics.

Subtypes	US (*n* = 36)	LMS-ST (*n* = 24)	MFS (*n* = 64)
FNCLCC grade 1	0 (0%)	1 (4.2%)	19 (29.7%)
FNCLCC grade 2	3 (8.3%)	9 (37.5%)	20 (31.3%)
FNCLCC grade 3	33 (91.7%)	14 (58.3%)	25 (39.1%)

Values are presented as number (%) or median (range).

**Table 3:** Survival probabilities across demographic and clinicopathologic subgroups.

OS	1-year	3-year	5-year	10-year
Total (*n* = 124)	97.6 (94.9–100.0)	90.5 (85.3–96.0)	88.3 (82.5–94.5)	77.9 (68.0–89.2)
Sex				
Female	100	93.6 (86.9–100.0)	93.6 (86.9–100.0)	80.1 (63.6–100.0)
Male	95.7 (91.0–100.0)	88.1 (80.7–96.2)	84.3 (75.8–93.9)	75.4 (63.5–89.6)
Age (year)				
<40	100	92.3 (78.9–100.0)	92.3 (78.9–100.0)	83.1 (64.1–100.0)
40–49	100	85.7 (69.2–100.0)	85.7 (69.2–100.0)	85.7 (69.2–100.0)
50–59	100	96.4 (89.8–100.0)	96.4 (89.8–100.0)	84.4 (64.3–100.0)
60<	95.3 (90.3–100.0)	88.4 (80.6–96.9)	84.0 (74.8–94.3)	70.5 (55.4–89.9)
Subtypes				
LMS-ST	100	85.9 (72.3–100.0)	85.9 (72.3–100.0)	75.2 (54.9–100.0)
MFS	96.9 (92.7–100.0)	95.2 (90.1–100.0)	93.2 (87.0–99.9)	83.8 (72.2–97.2)
US	97.1 (91.8–100.0)	84.2 (72.4–98.0)	80.2 (67.0–96.0)	66.8 (44.8–99.7)
FNCLCC_GRADE				
1	100	100	100	100
2	100	96.8 (90.8–100.0)	96.8 (90.8–100.0)	92.7 (83.5–100.0)
3	95.8 (91.2–100.0)	84.7 (76.3–93.9)	80.6 (71.1–91.3)	55.4 (36.4–84.4)
S-NCC	1-year	3-year	5-year	10-year
Total (n = 124)	95.9 (92.4–99.5)	89.2 (83.5–95.2)	85.4 (78.7–92.7)	76.3 (65.1–89.5)
Sex				
Female	98.1 (94.4–100.0)	91.1 (83.1–99.9)	91.1 (83.1–99.9)	81.0 (63.2–100.0)
Male	94.1 (88.7–99.9)	87.7 (80.1–96.1)	81.2 (71.5–92.2)	72.5 (58.6–89.6)
Age (year)				
<40	92.3 (78.9–100.0)	92.3 (78.9–100.0)	92.3 (78.9–100.0)	82.1 (62.1–100.0)
40–49	92.3 (78.9–100.0)	84.6 (67.1–100.0)	84.6 (67.1–100.0)	84.6 (67.1–100.0)
50–59	100	91.7 (81.3–100.0)	91.7 (81.3–100.0)	91.7 (81.3–100.0)
60-	95.2 (90.0–100.0)	87.7 (79.6–96.8)	80.1 (69.4–92.4)	64.7 (46.2–90.6)
Subtypes				
LMS-ST	95.5 (87.1–100.0)	83.5 (67.9–100.0)	83.5 (67.9–100.0)	71.6 (49.6–100.0)
MFS	95.2 (90.1–100.0)	95.2 (90.1–100.0)	90.6 (82.9–99.0)	80.3 (65.5–98.4)
US	97.2 (92.0–100.0)	80.8 (68.0–96.0)	76.5 (62.5–93.7)	76.5 (62.5–93.7)
FNCLCC_GRADE				
1	100	100	100	100
2	96.7 (90.5–100.0)	96.7 (90.5–100.0)	92.1 (81.9–100.0)	92.1 (81.9–100.0)
3	94.3 (89.1–99.9)	82.7 (73.8–92.6)	78.5 (68.6–89.8)	56.3 (36.3–87.3)
Presentation				
Primary	97.8 (94.8–100.0)	90.4 (84.3–97.0)	85.7 (78.1–94.1)	78.2 (66.3–92.3)
Recurred	91.7 (81.3–100.0)	86.6 (73.4–100.0)	86.6 (73.4–100.0)	72.1 (48.7–100.0)
Metastasis	80.0 (51.6–100.0)	80.0 (51.6–100.0)	80.0 (51.6–100.0)	80.0 (51.6–100.0)
BMI				
≤25	96.0 (91.7–100.0)	90.1 (83.3–97.4)	86.0 (77.7–95.2)	77.9 (65.5–92.6)
25<	95.4 (89.4–100.0)	87.6 (77.9–98.4)	84.5 (73.7–96.8)	72.4 (51.9–100.0)
Radiotherapy				
Yes	87.5 (72.7–100.0)	72.7 (52.9–100.0)	72.7 (52.9–100.0)	-
No	97.2 (94.0–100.0)	91.6 (86.1–97.4)	87.2 (80.2–94.7)	77.5 (65.8–91.3)
Chemotherapy				
Yes	90.9 (75.4–100.0)	68.2 (43.3–100.0)	68.2 (43.3–100.0)	-
No	96.3 (92.9–99.9)	91.0 (85.6–96.8)	86.9 (80.1–94.3)	80.2 (69.3–92.8)

Survival probability was expressed with 95% confidence interval (CI).

**Table 4:** Cox analysis of prognostic factors for OS and S-NCC.

		OS						S-NCC					
		Univariable Analysis			Multivariable Analysis			Univariable Analysis			Multivariable Analysis		
	*n* (%)	HR	95% CI	*p*-Value	HR	95% CI	*p*-Value	HR	95% CI	*p*-Value	HR	95% CI	*p*-Value
Total (n = 124)	124(100)												
Sex													
Male	69 (55.6)	2.07	0.75–5.72	0.162				1.94	0.70–5.36	0.205			
Female	55 (44.4)	(ref)						(ref)					
Subtypes													
US	36 (29.0)	2.79	1.01–7.76	0.0488	1.01	0.37–2.87	0.9846	2.86	1.02–7.99	0.045	1.21	0.41–3.52	0.7299
LMS-ST	24 (19.4)	2.14	0.68–6.77	0.1938	1.24	0.38–3.76	0.7083	2.05	0.65–6.51	0.224	1.14	0.35–3.75	0.8318
MFS	64 (51.6)	(ref)			(ref)			(ref)			(ref)		
FNCLCC_GRADE													
3	72 (58.1)	11.37	2.59–49.92	0.0013	10.87	2.32–50.80	0.0024	9.31	2.14–40.5	0.003	8.29	1.78–38.62	0.0071
1 or 2	52 (41.9)	(ref)			(ref)			(ref)			ref		
Presentation													
Metastasis	7 (5.6)	0.74	0.10–5.64	0.773				1.25	0.16–9.54	0.829			
Recurred	24 (19.4)	0.8	0.26–2.42	0.691				1.32	0.44–4.02	0.621			
Primary	93 (75.0)	(ref)						(ref)					
BMI *													
>25	45 (36.3)	0.94	0.37–2.35	0.889				0.9	0.36–2.27	0.829			
≤25	78 (62.9)	(ref)						(ref)					
Radiotherapy													
Yes	16 (12.9)	1.53	0.51–4.60	0.448				2.45	0.80–7.53	0.118			
No	108 (87.1)	(ref)						(ref)					
Chemotherapy													
Yes	13 (10.5)	2.31	0.76–7.02	0.138				4.16	1.34–12.88	0.014	3.68	1.18–11.50	0.0251
No	111 (89.5)	(ref)						(ref)			(ref)		

All *p*-values are based on Wald test, except for ‘FNCLCC_GRADE’, which was analyzed using Firth’s penalized likelihood test. * BMI missing in 1 patient.

**Table 5:** Univariable Cox analysis of prognostic factors for OS and S-NCC in FNCLCC grade 3.

			OS			S-NCC	
	n (%)	HR	95% CI	*p*-Value	HR	95% CI	*p*-Value
Total (*n* = 72)	72 (100)						
Sex							
Male	42 (58.3)	1.04	(0.35–3.08)	0.94	0.98	(0.33–2.92)	0.966
Female	30 (41.7)	(ref)			(ref)		
Subtypes							
US	33 (45.8)	0.99	(0.34–2.86)	0.981	1.15	(0.40–3.34)	0.791
LMS-ST	14 (19.4)	1.01	(0.28–3.68)	0.983	1	(0.27–3.61)	0.994
MFS	25 (34.7)	(ref)			(ref)		
Presentation							
Metastasis	2 (2.8)	0.88	(0.01–6.92)	0.9288	2.52	(0.02–19.83)	0.577
Recurred	10 (13.9)	2.06	(0.61–5.85)	0.2249	3.34	(0.99–9.43)	0.0511
Primary	60 (83.3)	(ref)			(ref)		
BMI							
>25	27 (38.0)	0.86	(0.32–2.31)	0.772	0.91	(0.34–2.44)	0.85
≤25	44 (62.0)	(ref)			(ref)		
Radiotherapy							
Yes	10 (13.9)	1.45	(0.41–5.16)	0.569	2.4	(0.66–8.69)	0.184
No	62 (86.1)	(ref)			(ref)		
Chemotherapy							
Yes	8 (11.1)	2.65	(0.84–8.42)	0.0976	4.32	(1.37–13.69)	0.0128
No	64 (88.9)	(ref)			(ref)		
Before HT							
Yes	25 (34.7)	0.71	(0.26–1.94)	0.507	0.75	(0.28–2.02)	0.566
No	47 (65.3)	(ref)			(ref)		
After HT							
Yes	2 (2.8)	1.11	(0.01–8.37)	0.9432	0.92	(0.01–6.89)	0.953
No	70 (97.2)	(ref)			(ref)		
HT							
Yes	28 (38.9)	0.62	(0.23–1.69)	0.353	0.63	(0.24–1.71)	0.367
No	44 (61.1)	(ref)			(ref)		
DM							
Yes	14 (19.4)	0.73	(0.16–3.19)	0.671	0.88	(0.20–3.96)	0.868
No	58 (80.6)	(ref)			(ref)		
TB							
Yes	4 (5.6)	3.27	(0.72–14.76)	0.124	2.79	(0.62–12.48)	0.179
No	68 (94.4)	(ref)			(ref)		
Other Cancer							
Yes	6 (8.3)	2.96	(0.83–10.52)	0.0941	3.92	(1.08–14.23)	0.0381
No	66 (91.7)	(ref)			(ref)		
Sarcoma							
Yes	2 (2.8)	4.25	(0.54–33.71)	0.171	4.37	(0.56–34.33)	0.16
No	70 (97.2)	(ref)			(ref)		
non-sarcoma							
Yes	4 (5.6)	2.37	(0.53–10.54)	0.258	3.29	(0.73–14.81)	0.121
No	68 (94.4)	(ref)			(ref)		
BPH							
Yes	4 (5.6)	5.87	(1.63–21.21)	0.0069	12.15	(3.17–46.52)	0.0003
No	68 (94.4)	(ref)			(ref)		

CI, confidence interval; HR, hazard ratio; BMI, body mass index; HT, hypertension; DM, diabetes mellitus; TB, tuberculosis; BPH, benign prostate hyperplasia; Other Cancer includes sarcoma and non-sarcoma.

## Data Availability

The data that support the findings of this work are unavailable due to privacy or ethical restrictions.

## Supplementary Materials

The following supporting information can be downloaded at: https://www.mdpi.com/article/10.3390/medicina62020271/s1, Table S1: Baseline clinical characteristics of patients (*n* = 124); Table S2: Cox analysis of prognostic factors for OS and S-NCC.
