# Supplementary material for: Clinical Characteristics of Complex Karyotype Soft Tissue Sarcomas: A Single-Institution Cohort Study
Source: Medicina (Kaunas). 2026 Jan 27;62(2):271. doi: 10.3390/medicina62020271 (PMC12941814; doi:10.3390/medicina62020271)
Supplement: Supplementary file 1 [file medicina-62-00271-s001.zip › medicina-4069508-supplementary.pdf]

Supplementary Table ST1. Baseline clinical characteristics of patients (n=124)

Subtype	Presentation	Anatomical_site
LMS-ST*	Primary	Long bones of lower limb
MFS	Primary	Upper limb
LMS-ST	Recurred	Lower limb
LMS-ST	Metastasis	Lower limb
MFS	Primary	Lower limb
MFS	Primary	Lower limb
LMS-ST	Primary	Lower limb
MFS	Primary	Upper limb
MFS	Primary	Lower limb
US-UPS	Primary	Lower limb
LMS-ST	Primary	Lower limb
MFS	Primary	Pelvis
MFS	Primary	Upper limb
US-UPS	Primary	Lower limb
US-UPS	Primary	Lower limb
LMS-ST	Recurred	Lower limb
US-UPS	Primary	Lower limb
MFS	Primary	Upper limb
MFS	Primary	Lower limb
LMS-ST	Recurred	Lower limb
LMS-ST	Metastasis	Lower limb
LMS-ST	Metastasis	Lower limb
MFS	Primary	Upper limb
MFS	Primary	Back
US-URS	Primary	Back
MFS	Primary	Upper limb
US-UPS	Recurred	Pelvis
MFS	Primary	Lower limb
MFS	Primary	Lower limb
US-UPS	Primary	Upper limb
MFS	Primary	Lower limb
MFS	Primary	Lower limb
MFS	Primary	Lower limb
LMS-ST	Primary	Lower limb
MFS	Primary	Lower limb
MFS	Primary	Lower limb
LMS-ST	Primary	chest wall
US-USS	Recurred	Pelvis
MFS	Primary	chest wall
US-UPS	Primary	Lower limb
MFS	Primary	Upper limb
MFS	Primary	Lower limb
MFS	Primary	Lower limb
MFS	Primary	Back
MFS	Recurred	Lower limb
MFS	Recurred	Lower limb
MFS	Recurred	Lower limb
MFS	Primary	Upper limb
US-USS	Primary	Upper limb
MFS	Recurred	Lower limb
LMS-ST	Primary	Pelvis
US-UPS	Recurred	chest wall
US-UPS	Recurred	Lower limb
US-UPS	Primary	Lower limb
MFS	Recurred	upper limb
LMS-ST	Primary	Lower limb
MFS	Primary	Lower limb
LMS-ST	Primary	Back
MFS	Primary	Pelvis
MFS	Primary	chest wall
MFS	Primary	upper limb
US-UES	Primary	Back
MFS	Primary	Pelvis
MFS	Recurred	Back
MFS	Primary	Lower limb
US-UPS	Primary	chest wall
US-UPS	Recurred	Back

MFS	Primary	Upper limb
MFS	Recurred	Lower limb
US-UPS	Metastasis	Abdominal wall
LMS-ST	Primary	Lower limb
US-UPS	Primary	Lower limb
MFS	Primary	chest wall
US-UPS	Primary	Pelvis
US-UPS	Primary	Lower limb
MFS	Primary	Upper limb
LMS-ST	Primary	Upper limb
US-UPS	Primary	Pelvis
US-UPS**	Primary	Long bones of lower limb
MFS	Primary	Upper limb
US-UPS	Primary	Lower limb
MFS	Recurred	Lower limb
US-UPS	Primary	Pelvis
MFS	Metastasis	Lower limb
MFS	Primary	Upper limb
MFS	Primary	Abdominal wall
LMS-ST	Primary	Lower limb
US-UPS	Primary	Lower limb
US-UPS	Primary	Lower limb
MFS	Primary	Lower limb
US-UPS	Primary	Lower limb
US-UPS	Primary	Back
US-UPS	Primary	Lower limb
MFS	Primary	Lower limb
US-USS	Recurred	Lower limb
MFS	Primary	Lower limb
US-USS	Primary	Lower limb
LMS-ST	Recurred	Pelvis
LMS-ST	Primary	Lower limb
MFS	Primary	Pelvis
MFS	Primary	Upper limb
LMS-ST*	Metastasis	pelvis
LMS-ST	Primary	Lower limb
MFS	Primary	Lower limb
MFS	Recurred	Upper limb
LMS-ST	Primary	Lower limb
MFS	Recurred	Upper limb
US-USS	Recurred	Abdominal wall
US-USS	Primary	Upper limb
MFS	Primary	Upper limb
MFS	Primary	Upper limb
MFS	Primary	Back
MFS	Recurred	Lower limb
US-UPS	Primary	Lower limb
MFS	Recurred	Lower limb
MFS	Primary	Upper limb
MFS	Primary	Upper limb
US	Primary	chest wall
MFS	Primary	Lower limb
MFS	Primary	Upper limb
LMS-ST	Primary	Upper limb
MFS	Recurred	Lower limb
LMS-ST	Metastasis	Lower limb
US-USS	Primary	Upper limb

*Two patients diagnosed with primary leiomyosarcoma of bone were identified.
**One patient with primary UPS of bone was included.

Supplementary Table ST2. Cox analysis of prognostic factors for OS and S-NCC.

		OS					
		**Multivariable analysis***			**Multivariable analysis †**		
	**n (%)**	**HR**	**(95% CI)**	**p-value**	**HR**	**(95% CI)**	**p-value**
Subtypes							
US	36 (29.0)	1.01	(0.37-2.87)	0.9846	1.09	(0.38-3.14)	0.8791
LMS	24 (19.4)	1.24	(0.38-3.76)	0.7083	1.28	(0.39-4.21)	0.6840
MFS	64 (51.6)	(ref)			(ref)		
FNCLCC_GRADE							
3	72 (58.1)	10.87	(2.32-50.80)	0.0024	10.77	(2.30-50.34)	0.0025
1 or 2	52 (41.9)	(ref)			ref		

		S-NCC					
		**Multivariable analysis***			**Multivariable analysis †**		
	**n (%)**	**HR**	**(95% CI)**	**p-value**	**HR**	**(95% CI)**	**p-value**
Subtypes							
US	36 (29.0)	1.21	(0.41-3.52)	0.7299	1.21	(0.41-3.52)	0.7328
LMS	24 (19.4)	1.14	(0.35-3.75)	0.8318	1.09	(0.32-3.71)	0.8877
MFS	64 (51.6)	(ref)			(ref)		
FNCLCC_GRADE							
3	72 (58.1)	8.29	(1.78-38.62)	0.0071	8.36	(1.79-39.06)	0.0069
1 or 2	52 (41.9)	ref			ref		
Chemotherapy							
Yes	13 (10.5)	3.68	(1.18-11.50)	0.0251	3.67	(1.17-11.50)	0.0259
No	111 (89.5)	(ref)			(ref)		

*included prospective cohort only (time era variable) as a confounding variable

† Included all cohort as a confounding variable
